# Exploratory Evaluation of Potential CRP and Ferritin Thresholds and Survival in Critically Ill Patients: A Pilot Prospective ICU Study

**DOI:** 10.3390/jcm14228172

**Published:** 2025-11-18

**Authors:** Manuel Cruz-Garcinuño, Antonio Martínez-Sabater, Ana Cobos-Rincón, Michał Czapla, Carmen Sarmiento-Iglesias, Enrique Polo-Andrade, Paula Álvarez, Antonio Rodríguez-Calvo, Urko Aguirre, Clara Isabel Tejada-Garrido, Raúl Juárez-Vela, Manuel Quintana-Diaz

**Affiliations:** 1 Doctoral Program in Medicine and Surgery, Autonomous University of Madrid, 28029 Madrid, Spain; manuel.delacruz01@estudiante.uam.es; 2 Department of Nursing, Faculty of Health Sciences, Research Group in Care (GRUPAC), University of La Rioja, 26004 Logroño, Spain; antonio.martinez-sabater@uv.es; 3 Nursing Care and Education Research Group (GRIECE), GIUV2019-456, Nursing Department, Universitat de València, 46010 València, Spain; michal.czapla@umw.edu.pl; 4 Faculty of Health Sciences, GRUPAC, University of La Rioja, 26004 Logroño, Spain; clara-isabel.tejada@unirioja.es (C.I.T.-G.); raul.juarez@unirioja.es (R.J.-V.); 5 Department of Emergency Medical Service, Division of Scientific Research and Innovation in Emergency Medical Service, Faculty of Nursing and Midwifery, Wroclaw Medical University, 51-616 Wrocław, Poland; 6 Intensive Care Unit, La Paz University Hospital, 28046 Madrid, Spain; maria.sarmiento@salud.madrid.org (C.S.-I.); enriquerafael.polo@saludmadrid.org (E.P.-A.); palvarezh@salud.madrid.org (P.Á.); manuel.quintana@uam.es (M.Q.-D.); 7 Department of Anesthesiology and Resuscitation, University Hospital of Salamanca (CAUSA), 37007 Salamanca, Spain; 8 Research Unit, Osakidetza Basque Health Service, Barrualde-Galdakao Integrated Health Organization, Galdakao-Usansolo Hospital, Kronikgune Institute for Health Services Research, 48903 Barakaldo, Spain; urko.aguirrelarracoechea@osakidetza.eus; 9 IdiPaz, La Paz Health Research Institute, Patient Blood Management Group, 28046 Madrid, Spain

**Keywords:** iron deficiency, ferritin, C-reactive protein, critical illness, Intensive Care Unit

## Abstract

**Background/Objectives:** Iron deficiency is a common condition in the general population, with a higher incidence in critically ill patients. Anemia associated with these alterations is linked to increased morbidity and mortality in ICU patients. This pilot study explores whether provisional CRP and ferritin thresholds might relate to survival, and examines the preliminary associations with analytical and clinical variables in critically ill patients. **Methods:** A prospective, observational pilot study was conducted on 75 ICU patients over three months. Hematological and biochemical parameters (CRP, ferritin, iron, transferrin, hemoglobin) were analyzed at admission, 48 h, and on days 4 and 7. Clinical data included age, sex, ICU stay, survival, SOFA and APACHE II scores, complications (AKI, acute lung injury), and interventions (mechanical ventilation, infections). Data were analyzed using mixed regression models and Wilcoxon tests. **Results:** In this pilot cohort (mean age 53.65 years; 61.33% male), survival was 82.67%. Higher CRP and ferritin levels were observed among non-survivors and those with AKI *p* < 0.05. A CRP level ≥145 mg/L was associated with a constellation of more unfavorable clinical indicators (older age, longer ICU stay, higher APACHE II and SOFA scores, more mechanical ventilation, higher AKI and infection rates, and reduced survival) *p* < 0.05. Ferritin levels were higher in males and non-survivors and showed positive correlations with SOFA score and ICU length of stay. The exploratory prognostic performance of the CRP threshold was AUC = 0.8103. **Conclusions:** Elevated CRP and ferritin concentrations were associated with reduced survival probability and indicators of greater clinical severity in critically ill patients. The provisional thresholds identified in this pilot study (CRP ≥ 145 mg/L, ferritin ≥ 300 ng/mL) may facilitate early risk stratification; however, these findings remain exploratory and require validation in larger, multicenter cohorts before clinical translation.

## 1. Introduction

Iron deficiency is a prevalent condition in the general population, but its incidence rises markedly in patients admitted to Intensive Care Units (ICUs) [1]. Critical illnesses exacerbate this deficiency through functional iron dysregulation, characterized by decreased iron absorption, impaired recycling, and reduced bioavailability of stored iron. In critically ill patients, anemia severity—often assessed by parameters such as hemoglobin and hematocrit—can significantly compromise oxygen transport, making anemia one of the primary contributors to morbidity and mortality during ICU stays [2,3].

Critical illnesses induce profound alterations in iron metabolism, largely mediated by the hormone hepcidin, a 25-amino acid polypeptide synthesized by hepatocytes. Hepcidin secretion is stimulated by inflammatory cytokines and iron overload. Once released, hepcidin binds to ferroportin, a cellular iron exporter found in duodenal enterocytes, macrophages, and hepatocytes, leading to its degradation. This process reduces dietary iron absorption, limits the release of recycled iron from macrophages, and lowers circulating iron levels, promoting the sequestration of iron in storage sites within hepatocytes [4].

Emerging evidence suggests that measuring hepcidin levels could help guide intravenous iron therapy in the patients most likely to benefit from this intervention. Other acute-phase reactants, such as ferritin (an intracellular iron storage protein) and lactoferrin (an iron-binding glycoprotein), are similarly regulated by the inflammatory response. The higher affinity of ferritin and lactoferrin for iron, compared to transferrin (a circulating iron transporter and negative acute-phase reactant), creates a state of hypoferremia. While cytokines directly induce rapid changes in ferritin and transferrin levels, hepcidin remains the key regulator of iron metabolism, determining the severity and duration of iron restriction. This pattern—characterized by low serum iron, low transferrin, and high ferritin—is observed in approximately 75% of critically ill patients within the first three days of ICU admission [4,5].

The metabolic changes in iron associated with critical illnesses have two major clinical implications: First, diagnosing functional iron deficiency is challenging and cannot be reliably achieved using standard iron study parameters. Second, patients with persistent iron dysregulation are at increased risk of developing nosocomial infections, cognitive dysfunction, neuromuscular dysfunction, and cardiopulmonary complications. Persistent functional iron deficiency can lead to severe metabolic imbalances, where reduced iron availability impairs vital organ function.

Consequently, further research should clarify phenotype-guided iron management in critical illness (absolute vs. functional iron deficiency), including the timing and selection of candidates for intravenous iron, while recognizing that iron chelation is not a standard therapy in the ICU and cannot be recommended outside clinical trials [6,7].

Globally, studies have highlighted the role of biomarkers like ferritin in predicting outcomes in critically ill patients. For example, a study conducted by M. Elouadani in Morocco [8] revealed that elevated levels of CRP, ferritin, and procalcitonin were associated with severe and critical forms of COVID-19, as well as poor prognosis. This research emphasized the importance of clinical-biological scoring systems incorporating multiple biomarkers to standardize treatment practices and ensure appropriate prescriptions for COVID-19 patients. Similarly, a study by Deng et al. [9] analyzed 100 hospitalized COVID-19 patients in the ICU and found that ferritin levels at admission could serve as an independent predictor of hospital mortality. Median ferritin concentrations in the deceased group were approximately three times higher than in survivors (1722.25 μg/L vs. 501.90 μg/L, *p* < 0.01). Ferritin levels also correlated with other inflammatory cytokines. Logistic regression analysis confirmed ferritin as an independent predictor of hospital mortality, particularly in patients with elevated ferritin levels, with an adjusted odds ratio of 104.97 (95% CI: 2.63–4185.89; *p* = 0.013). Furthermore, ferritin demonstrated superior discriminative capacity, with an area under the ROC curve (AUC) of 0.776 (95% CI: 0.737–0.907), outperforming both procalcitonin and CRP. These findings underscore the need for studies to evaluate the diagnostic utility of ferritin and CRP, particularly their AUC, in Spanish ICUs.

This pilot study explored whether provisional CRP and ferritin thresholds might relate to survival and examined preliminary associations with analytical and clinical variables in critically ill patients.

## 2. Materials and Methods

This pilot, observational, and prospective study was conducted in the Polytrauma and Burn Unit of the Intensive Care Medicine Service of Hospital La Paz (Madrid, Spain) Participants were prospectively enrolled from a cohort of patients admitted to this unit over 3 months (from 1 March, April, and 30 May 2024), following the ethics committee protocol Hospital Universitario La Paz (Madrid) Number PI-6034, date of approval 1 March 2024. Enrollment was carried out after obtaining informed consent, either directly from the patients or through their legal representatives.

The study included adult patients (over 18 years of age) admitted to the ICU for at least 7 days who provided informed consent. Patients with active hematological diseases or incomplete data (clinical or hematimetric) were excluded. A non-probabilistic convenience sampling method was applied, including all patients admitted to the unit who met the eligibility criteria.

Participants were followed until ICU discharge or death. Sample collection was performed following the unit’s protocol, which routinely monitors infection and inflammation markers. Data were collected at four time points: at admission, 48 h post-admission, and on days 4 and 7 of ICU stay. During the study period, 75 patients were admitted to the ICU. Two databases were created: one focusing on hematimetric variables (N = 44), referred to as the HEM-Database, and another including analytical variables such as CRP, procalcitonin, and others, encompassing the entire cohort (N = 75), referred to as the BIOCH-Database.

### Statistical Analysis

The characteristics of the participants were described using summary statistics. Frequencies and percentages were reported for categorical variables, while for continuous variables, means with standard deviations (SD) or medians with interquartile ranges (IQR) were used, as appropriate. The Mann–Whitney U test was used for non-parametric comparisons using STATA version 18 (StataCorp LLC, College Station, TX, USA). The following variables were collected: Sociodemographic Variables: Age, sex. Clinical Variables: ICU length of stay, survival status (yes/no), ICU admission Sequential Organ Failure Assessment (SOFA) score (ranging from 0 [low organ failure severity] to 24 [high organ failure severity]), and Acute Physiology and Chronic Health Evaluation APACHE II score (ranging from 0 [low acute illness severity] to 299 [high acute illness severity]. Complications: Acute Lung Injury (ALI) (yes/no), Acute Kidney Injury (AKI) (yes/no), Mechanical Ventilation: Use of mechanical ventilation (yes/no) and duration in days.

Infections: Diagnosis of infection (yes/no), infection site (pulmonary, abdominal, bloodstream, urinary, central nervous system, or other).

Hematological and Biochemical Parameters: Ferritina (ng/mL) Iron (μg/dL), IST (Iron Saturation Index, %): CRP (C-Reactive Protein, mg/L): Transferrin (mg/dL).

## 3. Results

Table 1 and Table 2 summarize the admission profile and clinical characteristics of ICU patients. Regarding reasons for admission, the most frequent were other types of burns (18.67%), followed by flame burns (13.33%) and septic shock or sepsis (8%). Other notable causes included multiple trauma (8%), traumatic brain injury (TBI) (5.33%), respiratory failure (4%), and other medical reasons (6.67%). The total number of patients included in the analysis was 75.

The demographic analysis revealed a mean age of 53.65 years (SD: 17.66), with a median of 57 years (range: 18–84). Most patients were male (61.33%), while 38.67% were female. The median ICU stay was 6 days, with a mean of 13.71 days (SD: 19.19) and a range of 1 to 86 days. Regarding survival outcomes, 82.67% of patients survived, while 17.33% did not.

Clinical scores indicated a mean APACHE II score of 12.37 (SD: 6.86) and a mean SOFA score of 3.74 (SD: 3.36). These scores reflect the severity of illness in the cohort, with interquartile ranges (IQR) of 7–16 for APACHE II and 1–6 for SOFA.

Complications were common among the patients. Acute lung injury was observed in 24% of cases, while 44% required mechanical ventilation, with a median duration of 0 days (mean: 6.59 days, SD: 13.43). Acute kidney injury (AKI) occurred in 25.68% of patients. Thromboembolic events (TDE) were less frequent, affecting 9.33% of the cohort.

Infections were a significant concern, as 41.33% of patients experienced at least one infection. Pulmonary infections were the most common (20%), followed by urinary infections (10.67%), bloodstream infections (9.33%), abdominal infections (8%), and central nervous system (CNS) infections (4%). Other types of infections were observed in 8% of patients.

Table 3 presents the descriptive characteristics of the biomarkers related to iron metabolism and inflammation, and Table 4 presents the mean, standard deviation, median, and interquartile range of the minimum values for the biochemical variables to provide an overview of survival. Ferritin (ng/mL) shows high variability in its values. The average values have a mean of 1037.01 ng/mL (SD = 1556.81), with an interquartile range of 322.45 to 1348.00 ng/mL. Iron (μg/dL) also shows variability. The minimum values have a mean of 37.86 μg/dL (SD = 34.28), ranging from 6.00 to 184.00 μg/dL, with a median of 25.00 μg/dL. The Iron Saturation Index (IST, %) shows minimum values with a mean of 22.28% (SD = 23.16), ranging from 4.00% to 111.00%. The average values have a mean of 34.14% (SD = 25.56), and the maximum values reach a mean of 51.26% (SD = 39.45), with a range of 8.00% to 159.00%. C-reactive protein (CRP, mg/L) shows minimum values with a mean of 13.74 mg/L (SD = 26.89), ranging from 0.50 to 182.60 mg/L, with a median of 4.80 mg/L. Transferrin (mg/dL) has a mean of 140.18 mg/dL (SD = 63.38), while the maximum values reach a mean of 165.84 mg/dL (SD = 69.50), with a range of 25.00 to 297.00 mg/dL.

Regarding the “HEM-Database,” aimed at establishing the relationship between ferritin, survival, and sex, the following statistically significant differences were identified: the minimum ferritin values are significantly different between women and men (*p* = 0.0467), with men presenting higher minimum ferritin values on average (602.81 ng/mL) compared to women (321.35 ng/mL). Regarding survival differences, the minimum ferritin values are significantly different based on survival status (*p* = 0.0064), with patients who did not survive having higher minimum ferritin values on average compared to those who survived. Similarly, the mean ferritin values are significantly different based on survival status (*p* = 0.0407), with patients who did not survive showing higher mean ferritin values (1971.20 ng/mL) compared to those who survived (725.61 ng/mL). Table 5 summarizes the HEM-Database analysis assessing the relationship between ferritin, survival, and sex.

To further analyze ferritin, the Mann–Whitney U test was performed to compare differences between two independent groups. The results revealed significant differences in multiple clinical and biochemical parameters. Men showed significantly higher minimum ferritin levels than women (*p* = 0.0467), reflecting differences in iron reserves. Patients who did not survive exhibited significantly higher minimum ferritin levels than survivors (z = 2.724, *p* = 0.0064). Regarding iron and infection, patients with infections had significantly higher minimum iron levels compared to those without infections (z = 3.153, *p* = 0.0012). Finally, concerning the Iron Saturation Index (IST), non-survivors showed significantly higher minimum IST levels than survivors (z = 2.132, *p* = 0.0321), while patients requiring mechanical ventilation had significantly higher maximum IST levels compared to those who did not require it (z = −2.120, *p* = 0.0336).

Concerning transferrin, patients with infections presented significantly higher minimum transferrin levels (z = 2.364, *p* = 0.0172), while patients requiring mechanical ventilation showed significant differences in transferrin levels (z = 2.545, *p* = 0.0109), suggesting alterations in iron metabolism in critically ill patients.

Regarding the Spearman correlation coefficient, it was used to evaluate the strength and direction of monotonic relationships between continuous variables. The following significant correlations were identified: A negative correlation (ρ = −0.4354, *p* = 0.0035) suggests that higher SOFA scores (indicative of greater organ dysfunction) were associated with lower minimum ferritin levels. A negative correlation (ρ = −0.4750, *p* = 0.0013) indicates that longer ICU stays were associated with lower minimum transferrin levels. Additionally, a negative correlation (ρ = −0.3974, ρ = −0.0083) suggests that prolonged mechanical ventilation was associated with lower transferrin levels. Table 6 summarizes additional analyses of ferritin and related biomarkers.

Finally, descriptive statistics were calculated to summarize the distribution of the main variables across different groups. These provide additional context to the findings. Non-survivors had a mean ferritin level of 1971.20 ng/mL (SD = 2650.80), significantly higher than survivors (mean = 725.61 ng/mL, SD = 812.97). Patients without infections had a higher mean iron level (59.00 μg/dL, SD = 40.56) compared to those with infections in “other” locations (38.02 μg/dL, SD = 10.38). The analysis compares two groups of patients based on the cutoff point of 300 for the variable ferropenia-300, with a sensitivity of 0.8182 and specificity of 0.7237. Group 0 includes patients with ferropenia300 < 300, while Group 1 includes patients with ferropenia 300 ≥ 300. Several clinical variables were analyzed to identify significant differences between the groups.

Regarding age, the mean was 55.80 years (SD: 19.48) in Group 0 and 56.42 years (SD: 16.57) in Group 1. The Wilcoxon test showed no significant differences (*p* = 0.953). Regarding sex, 50% of Group 0 were women compared to 29.17% in Group 1. The chi-square test also showed no significant differences (*p* = 0.158).

The duration of ICU stay was similar between groups, with a mean of 20.40 days (SD: 23.63) in Group 0 and 19.83 days (SD: 22.78) in Group 1. The Wilcoxon test indicated no significant differences (*p* = 0.822). However, a significant difference was observed in survival, where 10% of patients in Group 0 did not survive, compared to 37.50% in Group 1 (*p* = 0.036), suggesting that patients with ferropenia300 ≥ 300 have a higher proportion of non-survivors.

The Apache II score showed no significant differences (*p* = 0.705), with a mean of 12.90 (SD: 4.47) in Group 0 and 14.22 (SD: 7.70) in Group 1. Similarly, the SOFA score also showed no significant differences (*p* = 0.371), with a mean of 5.15 (SD: 3.27) in Group 0 and 4.22 (SD: 3.50) in Group 1. Regarding acute lung injury, 30% of Group 0 and 33.33% of Group 1 were affected, with no significant differences (*p* = 0.813).

Regarding the need for mechanical ventilation, 50% of Group 0 and 54.17% of Group 1 required it, with no significant differences (*p* = 0.783). The number of days on mechanical ventilation was also similar, with a mean of 9.95 days (SD: 16.65) in Group 0 and 10.00 days (SD: 16.22) in Group 1 (*p* = 0.861).

Other variables, such as acute kidney injury (AKI) (*p* = 0.423), thromboembolic disease (TDE) (*p* = 0.521), and infection (*p* = 0.467), showed no significant differences between groups. The location of the infection also showed no significant differences, including pulmonary location (*p* = 0.679), abdominal location (*p* = 0.261), urinary location (*p* = 0.521), CNS location (*p* = 0.356), blood location (*p* = 0.880), and other locations (*p* = 0.521).

**Figure 1:**
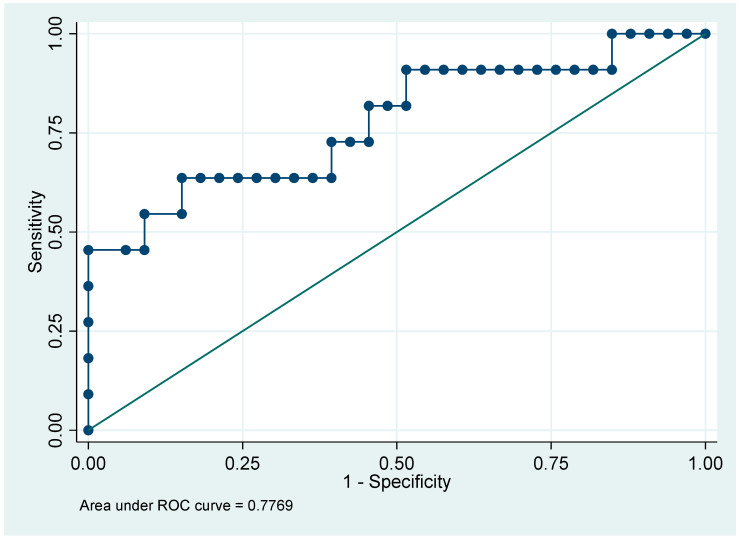
ROC Curve (Receiver Operating Characteristic) with a cutoff point of 300 for the variable ferropenia-300.

The only significant difference observed was in survival, where patients with ferropenia ≥ 300 (maximum value) had a higher proportion of non-survivors. No statistically significant differences were found in the other clinical variables. Regarding the ROC curve, the evaluated model has an AUC of 0.7769, indicating that it can correctly discriminate between positive and negative classes in approximately 78% of cases, which is considered good performance (Figure 1).

Regarding the “BIOCH-Database”, it was used to analyze C-reactive protein (CRP). Table 7 presents the results of two statistical analyses: the Wilcoxon tests (Rank-Sum Test) and Spearman correlations. In the Wilcoxon tests, which compare two groups in terms of a numerical variable such as CRP levels, it was observed that non-surviving patients had significantly higher mean CRP values compared to survivors (z = 3.360, *p* = 0.0005). Similarly, non-surviving patients showed significantly higher maximum CRP values compared to survivors (z = 3.489, *p* = 0.0003). Furthermore, patients with AKI had significantly higher maximum CRP values compared to those without AKI (z = −3.752, *p* = 0.0001).

As for the Spearman correlations, a moderate positive correlation was found between SOFA and maximum CRP (Rho = 0.5503, *p* = 0.0000), indicating that higher SOFA scores (an indicator of organ dysfunction) are associated with higher maximum CRP levels. Similarly, a moderate positive correlation was observed between days on mechanical ventilation and maximum CRP (Rho = 0.5206, *p* = 0.0000), suggesting that patients with higher CRP levels tend to require more days of mechanical ventilation. Additionally, a moderate positive correlation was identified between the Apache II score and maximum CRP (Rho = 0.4851, *p* = 0.0000), implying that patients with greater clinical severity have higher maximum CRP levels.

**Figure 2:**
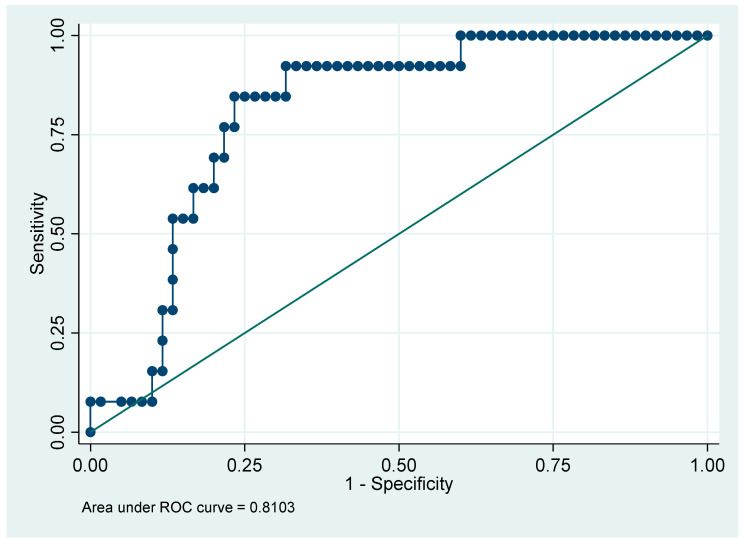
ROC Curve (Receiver Operating Characteristic) with a Cutoff point of CRP of 145 mg/L.

Table 8 presents the clinical characteristics and outcomes stratified by the maximum CRP cutoff of 145 mg/L. For continuous variables, the Wilcoxon rank-sum test was used to determine differences between groups, while for categorical variables, the Chi-square test was used to assess associations. Patients with CRP ≥ 145 were older, had longer ICU stays, higher Apache II and SOFA scores, greater need for mechanical ventilation, higher incidence of AKI and infections, and lower survival rates. The maximum CRP (maximum value) cutoff point of 145 effectively discriminates between patients with poorer prognoses and those with better clinical outcomes, as demonstrated by the AUC of 0.8103 (Figure 2).

## 4. Discussion

This pilot study explored whether provisional CRP and ferritin thresholds might relate to survival and examined preliminary associations with analytical and clinical variables in critically ill patients. It is well-established that hyperferritinemia is associated with high mortality, regardless of the underlying pathology [10,11,12]. In our study, the minimum ferritin values showed significant differences based on survival status (*p* = 0.0064), with higher average values observed in patients who did not survive. Similarly, the mean ferritin values were also significantly higher in non-survivors (1971.20 ng/mL) compared to survivors (725.61 ng/mL), with *p* = 0.0407.

Yang et al. [13] conducted a systematic review of 19 studies involving 125,490 patients. This review demonstrated that elevated serum iron levels are associated with an increased risk of mortality, particularly in sepsis patients. Regarding iron and infections, our results reveal that patients with infections had significantly higher minimum iron levels compared to those without infections (z = 3.153, *p* = 0.0012). This phenomenon may reflect a defense mechanism known as iron sequestration, in which the body reduces iron availability to limit pathogen growth. Therefore, our findings are consistent with those reported in Yang’s review.

With respect to CRP, our results are also consistent with the scientific literature. Chi et al. [14] analyzed 189 studies during the COVID-19 pandemic, confirming that elevated CRP levels are associated with worse clinical outcomes, including greater disease severity, ICU admission, and mortality (e.g., disease severity: SMD = −0.87 mg/L, 95% CI: [−1.27, −0.47], *p* < 0.001; mortality: SMD = −1.32 mg/L, 95% CI: [−1.95, −0.69], *p* < 0.001; ICU admission: SMD = −1.39 mg/L, 95% CI: [−1.68, −1.11], *p* < 0.001). In our study, mean CRP levels were significantly associated with survival, with a z/Rho of 3.360 and a *p*-value of 0.0005, indicating that non-survivors likely had higher mean CRP levels. Similarly, the maximum CRP levels showed an even stronger association with survival (z/Rho = 3.489, *p* = 0.0003). Both results are highly significant and suggest that both mean and peak CRP levels are strong prognostic indicators of survival.

Our findings align with those of Demelo-Rodríguez et al. [15], who identified a CRP cutoff of >5 mg/dL with moderate predictive capacity for mortality (AUC = 0.7). However, our study used a higher CRP cutoff (≥145 mg/L), which demonstrated better overall discriminative capacity (AUC = 0.8103), with high sensitivity (84.62%) and specificity (81.67%) for predicting adverse outcomes. Both studies conclude that elevated CRP levels are associated with worse clinical outcomes, and our data highlight an optimal cutoff (≥145 mg/L) with high sensitivity and specificity.

Regarding ICU predictors, Ríos-Toro et al. [16] highlighted that changes in SOFA scores between admission and day 2 were significantly associated with mortality (*p* = 0.002), identifying SOFA variations as an independent predictor of mortality (OR = 2.13, 95% CI: 1.18–3.86). Similarly, our data show significant differences in SOFA scores between the evaluated groups. In the group with CRP ≤ 145 mg/L, the mean score was 3.14 (SD: 3.15) and the median was 2.00 (IQR: 1.00–4.00), while in the group with CRP > 145 mg/L, the mean score was 5.15 (SD: 3.57) and the median was 5.00 (IQR: 2.00–8.50). The Wilcoxon analysis (z = −2.177) yielded a *p*-value of 0.0295, confirming that SOFA is a key indicator of clinical severity, and its relationship with other markers, such as CRP, may reflect the progression of organ dysfunction.

Regarding APACHE II, Ríos-Toro et al. [16] found that changes in APACHE II scores between admission and day 2 were significant in non-survivors (*p* = 0.001). A significant association was also identified between changes in APACHE II scores from admission to day 5 and mortality (*p* = 0.002). In our study, a moderate positive correlation was observed between average APACHE II scores and clinical severity. The group with CRP > 145 mg/L presented higher scores, which could indicate greater clinical severity compared to the group with CRP ≤ 145 mg/L. Both studies confirm that APACHE II is a robust marker of clinical severity, and its association with CRP may reflect disease progression in critically ill patients.

Regarding mechanical ventilation, our findings are consistent with those of Saldías-Peñafiel et al. [17], who reported that elevated CRP levels are significantly associated with a higher likelihood of requiring mechanical ventilation. In our study, elevated CRP levels were also significantly associated with mechanical ventilation, and patients with higher CRP levels required significantly more days of ventilation. Furthermore, Saldías-Peñafiel et al. noted that failure to reduce CRP levels in the early days predicts worse outcomes, which is consistent with our findings.

Finally, regarding ferritin, our results align with those of Liu et al. [18], who demonstrated that ferritin levels ≥ 300 ng/mL are associated with lower survival rates. In our study, non-survivors exhibited significantly higher minimum ferritin levels compared to survivors. Additionally, patients with infections showed significantly higher minimum iron levels than those without infections, reinforcing the hypothesis of iron sequestration as a defense mechanism. Our findings on ferritin’s predictive capacity are consistent with those reported by Deng et al. [9], who identified ferritin as an independent marker of in-hospital mortality in critically ill patients, with high discriminative capacity (AUC = 0.822). This pilot study has several limitations. First, the sample size (n = 75) is modest, which restricts statistical power and precision of estimates. The analyses were therefore intentionally limited to exploratory, primarily univariable assessments of associations between biomarker levels (CRP and ferritin), sociodemographic factors, and clinical variables, rather than definitive inference. Given the number of patients and events, proceeding with multivariable modeling was considered statistically unviable; with this cohort size, any model would likely be underpowered and at risk of overfitting while identifying few, if any, independent predictors. Accordingly, findings should be viewed as hypothesis-generating. We did not attempt to establish definitive CRP or ferritin thresholds; instead, we characterized patterns of association to inform future study design. Additional limitations include the single-center design, potential residual confounding (e.g., unmeasured chronic inflammatory conditions), and the absence of external validation or longitudinal repeated measures beyond the initial ICU assessments. These preliminary observations provide a foundation for larger, adequately powered multicenter studies in which robust multivariable adjustment and validation of independent predictive factors will be feasible. In addition, the infections were recorded as incident events. No determination of the causality of death was made due to the nature of the study.

## 5. Conclusions

In this pilot cohort, higher CRP and ferritin levels were associated with poorer survival and more adverse clinical indicators. Non-survivors showed elevated values, and the provisional thresholds CRP ≥ 145 mg/L and ferritin ≥ 300 ng/mL coincided with greater illness severity and longer ICU stay. CRP displayed exploratory discriminative performance AUC = 0.8103 (81%), while ferritin showed moderate discrimination AUC = 0.7769 (78%) These observations are hypothesis-generating and require validation in larger, adequately powered studies before any clinical implementation is considered.

## Figures and Tables

**Table 1:** Clinical and Sociodemographic Characteristics of the Sample (Categorical Variables).

Variable	Value/Frequency (n)	Percentage (%)
**Reason for Admission**		
- Septic Shock or Sepsis	6	8.00
- Flame Burn	10	13.33
- Multiple Trauma	6	8.00
- TBI (Traumatic Brain Injury)	4	5.33
- Respiratory Failure	3	4.00
- Burns (Other)	14	18.67
- Other Medical Reasons	5	6.67
- Total	75	100.00
**Sex**		
- Female	29	38.67
- Male	46	61.33
- Total	75	100.00
**Survival**		
- Yes	62	82.67
- No	13	17.33
- Total	75	100.00
**Acute Lung Injury**		
- Yes	18	24.00
- No	57	76.00
- Total	75	100.00
**Mechanical Ventilation**		
- Yes	33	44.00
- No	42	56.00
- Total	75	100.00
**Acute Kidney Injury (AKI)**		
- Yes	19	25.68
- No	55	74.32
- Total	74	100.00
**Thromboembolic Events (TDE)**		
- Yes	7	9.33
- No	68	90.67
- Total	75	100.00
**Infection**		
- Yes	31	41.33
- No	44	58.67
- Total	75	100.00
**Pulmonary Infection**		
- Yes	15	20.00
- No	60	80.00
- Total	75	100.00
**Abdominal Infection**		
- Yes	6	8.00
- No	69	92.00
- Total	75	100.00
**Urinary Infection**		
- Yes	8	10.67
- No	67	89.33
- Total	75	100.00
**Central Nervous System Infection**		
- Yes	3	4.00
- No	72	96.00
- Total	75	100.00
**Bloodstream Infection**		
- Yes	7	9.33
- No	68	90.67
- Total	75	100.00
**Other Infections**		
- Yes	6	8.00
- No	69	92.00
- Total	75	100.00

Note: Categorical variables are presented as absolute frequencies (n) and percentages (%).

**Table 2:** Clinical and Sociodemographic Characteristics of the Sample (Continuous Variables).

Variable	Mean (SD)	Median (P50)	Min–Max	Interquartile Range
Age (years)	53.65 (17.66)	57.00	18–84	41–68
ICU Stay (days)	13.71 (19.19)	6.00	1–86	4–12
APACHE II Score	12.37 (6.86)	11.00	1–29	7–16
SOFA Score	3.74 (3.36)	3.00	0–12	1–6
Mechanical Ventilation (days)	6.59 (13.43)	0.00	0–62	0–6

Note: Continuous variables include the mean, standard deviation (SD), median (P50), and interquartile ranges.

**Table 3:** Characteristics of different biomarkers related to iron metabolism and inflammation.

Parameter	N	Mean ± SD	Median [IQR]	Range
**Ferritin (ng/mL)**	44	1037.01 ± 1556.81	541.67 [322.45–1348.00]	44.00–9446.00
**Iron (μg/dL)**	44	56.61 ± 38.85	44.25 [32.81–63.50]	9.00–184.00
**IST (%)**	43	34.14 ± 25.56	24.00 [18.67–43.88]	8.00–111.00
**Transferrin (mg/dL)**	43	140.18 ± 63.38	130.11 [86.25–196.00]	25.00–284.00
**CRP (mg/L)**	73	96.05 ± 66.33	87.41 [34.58–148.48]	0.50–225.23

Note: Units: Ferritin (ng/mL), Iron (μg/dL), Transferrin (mg/dL), CRP (mg/L), IST (%). Continuous variables are presented as mean (standard deviation, SD), median (P50), and interquartile range (P25–P75). Valid N per biomarker is reported. Statistics presented: mean ± SD, median [interquartile range, IQR], and range (min–max). Note: Adult reference ranges: ferritin women 15–150 ng/mL, men 30–400 ng/mL; serum iron 60–170 μg/dL; transferrin saturation (TSAT/IST) 20–45%; transferrin 200–360 mg/dL; CRP < 5 mg/L. Ranges may vary by laboratory, analytical method, sex, and age.

**Table 4:** Standard Deviation, Median, and Interquartile Range of Minimum Values for Biochemical Variables.

Variable	N	Standard Deviation (SD)	Median (P50)	Interquartile Range (P25–P75)
Ferritin ng/mL–Minimum	44	597.34	319.50	128.00–496.50
Iron ug/dL–Minimum	44	34.28	25.00	17.00–45.50
IST (Iron Saturation Index) %–Minimum	43	23.16	16.00	10.00–23.00
CRP (C-Reactive Protein) mg/L–Minimum	73	26.89	4.80	0.50–13.60
Transferrin mg/dL–Minimum	43	67.61	124.00	67.00–173.00
Total–Minimum	247	308.06	25.00	9.00–104.00

Note: For each variable, N corresponds to the number of patients with at least one measurement. Values summarize per-patient minima (one per patient): standard deviation (SD), median (P50), and interquartile range (P25–P75).

**Table 5:** HEM-Database. Ferritin and Statistical Association.

	N	Median [P25–P75]	*p*-Value
**Minimum ferritin ng/mL by sex**			
**Female**	17	137.00 [67.00–458.00]	
**Male**	27	352.00 [224.00–1044.00]	0.0467 (*)
**Minimum ferritin ng/mL by survival**			
**No**	11	348.00 [NA]	
**Yes**	33	642.00 [NA]	0.0064 (**)
**Longitudinal mean ferritin ng/mL by survival**			
**No**	11	939.00 [570.82–2508.75]	
**Yes**	33	451.88 [212.67–968.00]	
**Total**	44	541.67 [322.45–1348.00]	0.0407 (*)

Notes: For each variable, N corresponds to the number of patients with at least one measurement. We report medians [P25–P75] per category, consistent with distribution skewness and the use of nonparametric tests (Rank-Sum). When P25–P75 are not available, “NA” is indicated. We explicitly define “minimum ferritin” as the lowest value recorded during the ICU stay, distinct from admission values. (*) Significant difference, *p* < 0.05, (**) Significant difference, *p* < 0.01.

**Table 6:** Combined Analysis (Mann–Whitney U Test and Spearman Correlations).

Test Type	Variable 1	Variable 2/Category	Group 1/Rho	Group 2/*p*	z/Significant	Significant
**Mann–Whitney U Test**	Ferritin (minimum)	Sex	Female	Male	−1.989	0.0467
	Ferritin (minimum)	Survival	No	Yes	2.724	0.0064
	Iron (minimum)	Infection	No	Yes	3.153	0.0012
	Iron (minimum)	Location (OTHERS)	No	Yes	2.035	0.0401
	TSI (minimum)	Survival	No	Yes	2.132	0.0321
	TSI (maximum)	Mechanical Ventilation	No	Yes	−2.120	0.0336
	Transferrin (minimum)	Infection	No	Yes	2.364	0.0172
**Spearman Correlations**	SOFA	Ferritin (minimum)	−0.4354 *	0.0035	-	Yes
	Days in ICU	Transferrin (minimum)	−0.4750	0.0013	-	Yes
	Days on MV	Transferrin (minimum)	−0.3974	0.0083	-	Yes

* We must consider the non-synchronous nature of minimum ferritin with respect to SOFA and the absence of causal inference in this pilot study. The different metrics (minimum vs. maximum vs. mean) capture different temporal facets of the inflammatory response, ICU = Intensive Care Unit, MV = Mechanical ventilation, TSI: Transferrin Saturation Index, SOFA = Sequential Organ Failure Assessment.

**Table 7:** Statistical Analysis by CRP.

Variable	Category/Variable 2	Group 1	Group 2	z/Rho	*p* (Exact)	Significant
**CRP (mean)**	Survival	No	Yes	3.360	0.0005	Yes
**CRP (maximum)**	Survival	No	Yes	3.489	0.0003	Yes
**CRP (maximum)**	AKI	No	Yes	−3.752	0.0001	Yes
**SOFA Score**	PCR (maximum)	-	-	0.5503	0.0000	Yes
**Days on MV**	PCR (maximum)	-	-	0.5206	0.0000	Yes
**Apache II Score**	PCR (maximum)	-	-	0.4851	0.0000	Yes

**Table 8:** Analysis Between the Cutoff of CRP of 145 mg/L.

Variable	Group CRP < 145 (0)	Group CRP ≥ 145 (1)	Statistical Test	*p*-Value
**Age (years)**	Mean: 50.87 (SD: 17.75) Median: 51.50 (37.00–64.50)	Mean: 59.71 (SD: 16.39) Median: 62.00 (50.00–74.00)	Wilcoxon (z = −1.951)	0.0511
**ICU Stay (days)**	Mean: 10.50 (SD: 15.19) Median: 5.00 (2.00–9.00)	Mean: 22.67 (SD: 25.53) Median: 11.00 (6.00–28.00)	Wilcoxon (z = −2.972)	0.0030
**Survival (%)**	Yes: 94.23% No: 5.77%	Yes: 52.38% No: 47.62%	Chi-square (χ^2^ = 17.8994)	<0.001
**Apache II Score**	Mean: 10.92 (SD: 6.29) Median: 10.00 (6.00–16.00)	Mean: 15.45 (SD: 6.57) Median: 14.00 (10.50–20.50)	Wilcoxon (z = −2.523)	0.0116
**SOFA Score**	Mean: 3.14 (SD: 3.15) Median: 2.00 (1.00–4.00)	Mean: 5.15 (SD: 3.57) Median: 5.00 (2.00–8.50)	Wilcoxon (z = −2.177)	0.0295
**Mechanical Ventilation (%)**	Yes: 34.62% No: 65.38%	Yes: 71.43% No: 28.57%	Chi-square (χ^2^ = 8.1842)	0.004
**Days on Mechanical Ventilation**	Mean: 4.29 (SD: 9.25) Median: 0.00 (0.00–2.50)	Mean: 12.90 (SD: 19.72) Median: 3.00 (0.00–12.00)	Wilcoxon (z = −2.894)	0.0038
**AKI (%)**	Yes: 17.65% No: 82.35%	Yes: 42.86% No: 57.14%	Chi-square (χ^2^ = 5.0420)	0.025
**Infection (%)**	Yes: 30.77% No: 69.23%	Yes: 66.67% No: 33.33%	Chi-square (χ^2^ = 7.9631)	0.005

## Data Availability

The data generated are available upon reasonable request from the corresponding authors.

## References

[1] Muñoz M., Leal-Noval S.R., García-Erce J.A., Naveira E. (2007). Prevalence and treatment of anemia in the critically ill patient. Med. Intensiv..

[2] Vincent J.L., Baron J.F., Reinhart K., Gattinoni L., Thijs L., Webb A., Meier-Hellmann A., Nollet G., Peres-Bota D., ABC (Anemia and Blood Transfusion in Critical Care) Investigators (2002). Anemia and blood transfusion in critically ill patients. JAMA.

[3] Tacke F., Nuraldeen R., Koch A., Strathmann K., Hutschenreuter G., Trautwein C., Strnad P. (2016). Iron parameters determine the prognosis of critically ill patients. Crit. Care Med..

[4] Litton E., Lim J. (2019). Iron metabolism: An emerging therapeutic target in critical illness. Crit. Care.

[5] Huang I., Pranata R., Lim M.A., Oehadian A., Alisjahbana B. (2020). C-reactive protein, procalcitonin, D-dimer, and ferritin in severe coronavirus disease-2019: A meta-analysis. Ther. Adv. Respir. Dis..

[6] Lasocki S., Pène F., Ait-Oufella H., Aubron C., Ausset S., Buffet P., Huet O., Launey Y., Legrand M., Lescot T. (2020). Management and prevention of anemia (acute bleeding excluded) in adult critical care patients. Ann. Intensive Care.

[7] Kell D.B., Pretorius E. (2014). Serum ferritin is an important inflammatory disease marker, as it is mainly a leakage product from damaged cells. Metallomics.

[8] Elouadani M., Konzi C., Addi Y., Mourji D., Biaz A., Elmechtani S., Ennibi K., Dami A., Bouhsain S. (2022). C-reactive protein, ferritin, and procalcitonin in 300 Moroccan patients with COVID-19. Clin. Lab..

[9] Deng F., Zhang L., Lyu L., Lu Z., Gao D., Ma X., Guo Y., Wang R., Gong S., Jiang W. (2021). Increased levels of ferritin on admission predict intensive care unit mortality in patients with COVID-19. Med. Clin. (Engl. Ed.).

[10] Carcillo J.A., Sward K., Halstead E.S., Telford R., Jimenez-Bacardi A., Shakoory B., Simon D., Hall M. (2016). A systemic inflammation mortality risk assessment contingency table for severe sepsis. Pediatr. Crit. Care Med..

[11] Garcia P.C., Longhi F., Branco R.G., Piva J.P., Lacks D., Tasker R.C. (2007). Ferritin levels in children with severe sepsis and septic shock. Acta Paediatr..

[12] Bennett T.D., Hayward K.N., Farris R.W., Ringold S., Wallace C.A., Brogan T.V. (2011). Very high serum ferritin levels are associated with increased mortality and critical care in pediatric patients. Pediatr. Crit. Care Med..

[13] Yang D.C., Zheng B.J., Li J., Yu Y. (2024). Iron and ferritin effects on intensive care unit mortality: A meta-analysis. World J. Clin. Cases.

[14] Chi L., Wang S., Wang X., Yang C., Luo J. (2023). Predictive value of C-reactive protein for disease severity and survival in COVID-19 patients: A systematic review and meta-analysis. Clin. Exp. Med..

[15] Demelo-Rodríguez P., Galeano-Valle F., Marcelo-Ayala A., Fernández-Carracedo E., Cuenca-Zarzuela A., Gómez-Morales M., Alvarez-Sala-Walther L.A., Bellón-Cano J.M., Del-Toro-Cervera J. (2020). C-reactive protein level predicts 30-day mortality and bleeding in patients with venous thromboembolism: A prospective single-center study. Med. Clin..

[16] Rios-Toro J.J., Pola-Gallego de Guzman M.D., Guerrero-Marin M., Rodriguez-Rubio D., Ruiz-Garcia M.I., Aguilar-Alonso E., Rivera-Fernandez R. (2021). Prognostic value of variations in serum biomarkers and prognostic score values between admission and second day in intensive care unit septic patients. Cureus.

[17] Saldías-Peñafiel F., Salinas-Rossel G., Farcas-Oksenberg K., Reyes-Sánchez A., Díaz-Patiño O. (2019). Usefulness of serum C-reactive protein in the diagnosis and treatment of immunocompetent adults hospitalized with community-acquired pneumonia. Rev. Med. Chil..

[18] Liu M., Yang C., Chu Q., Wang J., Wang Q., Kong F., Sun G. (2022). High serum levels of ferritin may predict poor survival and walking ability for patients with hip fractures: A propensity score matching study. Biomark. Med..

